# Productive, Morpho-Physiological, and Postharvest Performance of Six Basil Types Grown in a Floating Raft System: A Comparative Study

**DOI:** 10.3390/plants12030486

**Published:** 2023-01-20

**Authors:** Michele Ciriello, Valerio Cirillo, Luigi Formisano, Christophe El-Nakhel, Antonio Pannico, Stefania De Pascale, Youssef Rouphael

**Affiliations:** Department of Agricultural Sciences, University of Naples Federico II, 80055 Portici, Italy

**Keywords:** hydroponic, ion chromatography, leaf mass area, *Ocimum* L., shelf-life, water loss

## Abstract

Basil (*Ocimum* sp.) is one of the world’s most famous culinary fresh herbs, characterized by rapid growth that makes it particularly suitable for hydroponic cultivation. This study aimed to evaluate the adaptability of six types of basil to a closed-loop hydroponic system (floating raft system) and their post-harvest performance. Twenty-three days after transplantation, productivity, morpho-physiological performance, and mineral profile (by ion chromatography) were evaluated. At 3, 6, and 9 days after harvest, the loss of water from the from leaves stored at 10 °C in the dark was evaluated. Although the total fresh production of Thai, Mexican, and Genovese did not differ significantly, the latter provided a higher fresh leaf weight (16.52 g of plant^−1^) despite a lower leaf number (30.06 n. of plant^−1^). Nine days after harvest, Thai and Mexican showed the lowest water loss. Although Mexican Purple had the lowest net CO_2_ assimilation, it accumulated the highest concentration of ascorbic acid (909.41 mg 100 g fw^−1^).

## 1. Introduction

Although native to Asia (India, Pakistan, Iran, Thailand), subtropical Africa (where they grow wild), and South America, plants of the genus *Ocimum* L. are currently widespread and cultivated in many regions of the world [1,2]. These annual and perennial herbaceous aromatic plants, generally identified with the name basil, have previously been used for their therapeutic properties in folk medicine [3]. Currently, they are the undisputed stars of popular recipes in traditional cuisine and gourmet preparations, as well as in industrial derivative products such as dietary supplements, perfumes and soaps, cosmetics, and medicines [4]. The different uses to which these plants lend themselves result from inherent genetic variability; not surprisingly, the genus *Ocimum* L. includes more than 150 species [5]. The different species, in addition to diversifying in morphological characteristics (color and shape of leaves and flowers, etc.), are also distinguished by a different composition of the phytochemical profile (aromatic and phenolic) [6] induced by human interference in cultivation, selection, and hybridization [7]. However, the most essential and globally consumed species is *Ocimum basilicum* L., better known as sweet basil, among which the Genovese type has carved out a prestigious position. Its unique and unmistakable sensory characteristics, conferred by a mixture of monoterpenes and phenylpropanoids, have made it indispensable in traditional Italian dishes (Caprese salad and pizza Margherita) [8,9]. For processing purposes, basil is the crucial ingredient in “pesto sauce”, a renowned green condiment typical of the Liguria region (Italy) that has crossed national borders and become a “must-have” ready-to-eat product of world cuisine [10]. The need of the processing industry to obtain a product with standardized quality characteristics (i.e., color, aroma, and texture) has resulted in the gradual shift of Genovese basil cultivation from open field to protected cultivation. It is no coincidence that more than 50% of the Italian production of Genovese basil is produced in hydroponics, which guarantees high yields and better nutritional quality, as well as the deseasonalization of the production and the shortening of the production cycle [11,12,13]. Trials carried out in collaboration between researchers and producers have elected the floating raft system as the par excellence hydroponic system for the growing of Genovese basil, as it combines simplicity of management and excellent plant adaptability [14]. While the Genovese type has brought fame to the genus *Ocimum* L., it has also put other types and “minor species” on the culinary sidelines. The rise of gourmet cuisine, which mixes tradition and innovation, and the demand for healthy products in gastronomy and pharmaco-cosmetics are gradually driving the discovery of basil types and species with a not yet fully explored potential [15,16]. Examples include Thai and Mexican basils with their exotic and spicy flavors, Lemon basil with its citrus notes, and the red cultivars that stand out for their richness in anthocyanins [16,17]. However, their cultivation is relegated to specific geographic areas, and the limited interest of the scientific community has not allowed for a comprehensive assessment of their productive response to soilless cultivation. Little is known about their adaptability to alternative cropping systems that could be a springboard for these new and underexplored basil types. In addition, considering the possibility of seasonally adjusting production with hydroponic cultivation, it would be interesting to understand their production potential. Our work aimed to evaluate the productive, morpho-physiological of six basil types (*Ocimum basilicum* L. var thyrsiflora, *Ocimum basilicum* L. cv Cinnamon, *Ocimum × citriodorum*, *Ocimum basilicum* L. cv Italiano Classico, *Ocimum basilicum* L. cv Purple Ruffle, *Ocimum basilicum* L. cv Dark Opal) grown in floating raft system to provide the basis for their possible inclusion into the common cultivation processes of basil. Not least, considering that most studies on basil shelf-life have focused exclusively on the application of post-harvest treatments (e.g., application of high- or low-intensity light and influence of packaging) [18,19,20], in our work we also compared the shelf-life of six basil genotypes differing in morpho-anatomical characteristics. As far as we know, this is the first research investigating these aspects, establishing a basis for future studies.

## 2. Results

### 2.1. Yield and Biometric Indices

**Figure 1:**
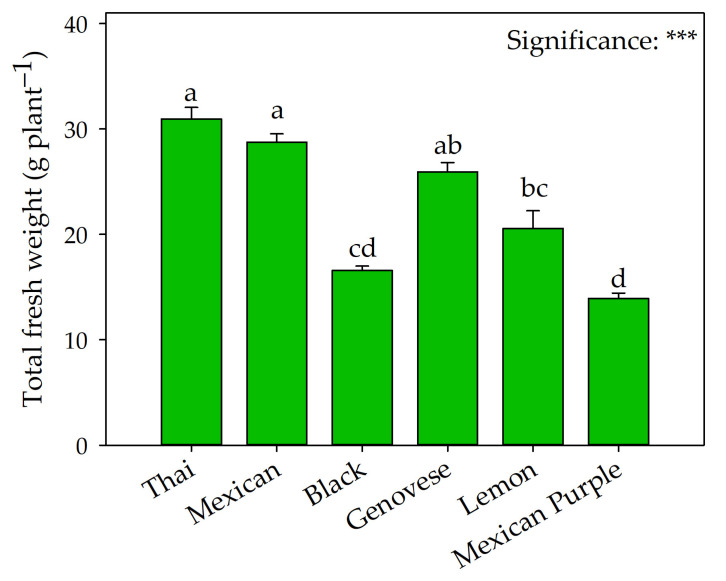
Comparison of Thai, Mexican, Black, Genovese, Lemon, and Mexican Purple basil on total fresh weight. Data are mean values ± standard error, *n* = 4. Mean comparisons were performed by Tukey HSD test. Different letters indicate significant mean differences. *** denote significant effects at *p* ≤ 0.001.

Except for root length, all yield and biometric parameters shown in Figure 1 and Table 1 were significantly affected by genetic material. Specifically, Black and Mexican Purple basil were less productive than Mexican, Thai, and Genovese (Figure 1). The latter was characterized by a higher leaf fw (16.52 g pt^−1^). As shown in Table 1, the highest stem fw, LMA, and leaf number were obtained by Mexican and Thai, with the latter showing the highest height (33.61 cm) and the lowest leaf-to-stem ratio fw that was not significantly different from that of Mexican. As for stem dm and total dm, the highest values were recorded in Lemon. Although Lemon also had the highest leaf dm values, these were not significantly different from Thai and Black ones. In contrast, Black and Mexican Purple had the lowest root dw and total dw values.

Although Mexican Purple and Black did not differ significantly in leaf dw and stem dw, the former had the lowest values. Regarding leaf area, Mexican Purple showed values about 1.5 times lower than Mexican.

### 2.2. Morpho-Physiological Traits

**Figure 2:**
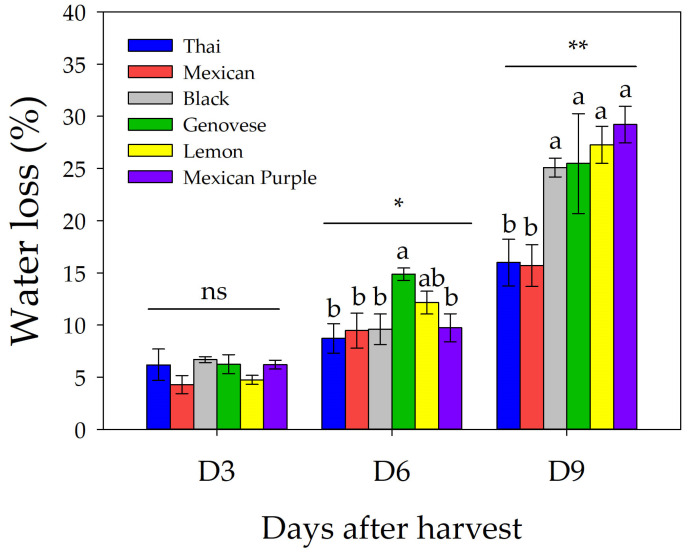
Comparison of Thai, Mexican, Black, Genovese, Lemon, and Mexican Purple basil on water loss percentage measured after three (D3), six (D6) and nine (D9) days after 10 °C storage. Data are mean values ± standard error, *n* = 5. Mean comparisons were performed by Tukey HSD test within each time point. Different letters indicate significant mean differences. Asterisks denote significant effects according to ANOVA within each date (ns = nonsignificant; * = *p* ≤ 0.05; ** = *p* ≤ 0.01).

As shown in Figure 2, water loss did not differ significantly on the third day of storage (D3) regardless of basil type; in contrast, a significant difference was observed on the sixth and ninth days of storage (D6 and D9) with *p* < 0.05 and 0.01, respectively. Relative to D6, the highest absolute percentage of water loss was recorded in Genovese, although it did not differ significantly from Lemon. At D9, the lowest water loss values were recorded in Thai and Mexican, while Black, Genovese, Lemon, and Mexican Purple basil did not differ significantly.

As shown in Table 2, statistical analysis indicated significant differences for E, A_CO2_, and WUEi. The lowest A_CO2_ and WUEi were recorded in Mexican Purple. As for E, the values recorded in Mexican were about 1.2 times higher than those in Genovese.

The pigments reported in Table 3 were significantly influenced by genetic material. Nevertheless, all pigments were statistically similar between Thai and Mexican and Black and Mexican Purple. In contrast, compared to Genovese, chlorophyll a, b, and total were significantly higher in Lemon, while an opposite trend was observed for carotenoids.

### 2.3. Ascorbic Acid Concentration and Minerals

**Figure 3:**
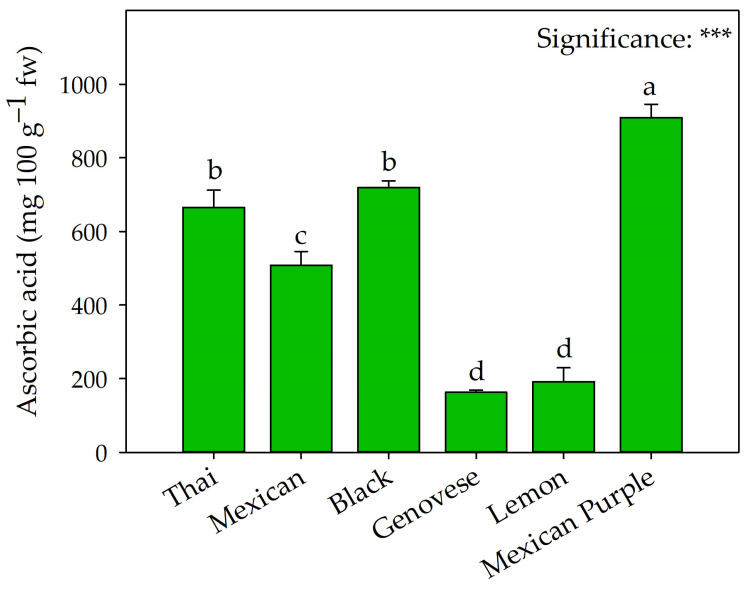
Comparison of Thai, Mexican, Black, Genovese, Lemon, and Mexican Purple basil on ascorbic acid concentration. Data are mean values ± standard error, *n* = 4. Mean comparisons were performed by Tukey HSD test. Different letters indicate significant mean differences. *** denote significant effects at *p* ≤ 0.001. fw: fresh weight.

**Figure 4:**
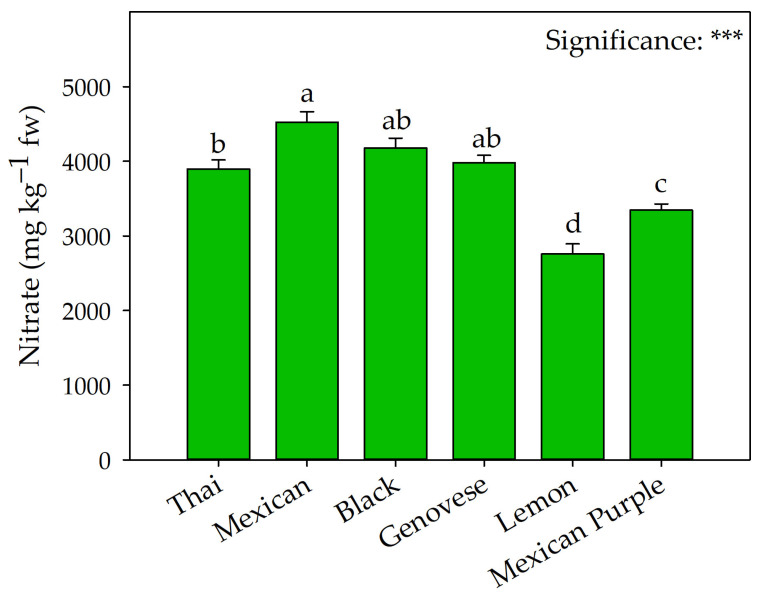
Comparison of Thai, Mexican, Black, Genovese, Lemon, and Mexican Purple basil on nitrate concentration. Data are mean values ± standard error, *n* = 4. Mean comparisons were performed by Tukey HSD test. Different letters indicate significant mean differences. *** denote significant effects at *p* ≤ 0.001. fw: fresh weight.

As shown in Figure 3, the highest ascorbic acid values were recorded in Mexican Purple, while the lowest were in Genovese and Lemon. The latter was also characterized by the lowest nitrate concentration (Figure 4).

All mineral elements reported in Table 4 were significantly affected by genetic material with a *p* < 0.001. Among all minerals, K was the most abundant, ranging from 51.53 g kg^−1^ dw (Mexican) to 35.50 g kg^−1^ dw (Lemon). The highest Cl values were observed in the Black and Mexican Purple cultivars. The latter was also characterized by the lowest values of P. The concentration of Ca and Mg did not differ between Black and Genovese; in contrast, Genovese showed a higher concentration of Na.

## 3. Discussion

### 3.1. Underexplored Basil Varieties Are Suitable for Floating Cultivation

The aromatic plants of the genus *Ocimum* L. show a wide genetic variability in morphological and qualitative characteristics, resulting from extensive selection by breeders and pressure from climatic factors, as they are geographically spread over vast and different areas of the world [21,22]. The morphological traits and the fresh yield of the six basil types differed significantly (Figure 1 and Table 1); this result can be attributed only to genetics since the basil were grown under the same environmental and growth conditions. Specifically, the hydroponic system used (floating raft system) significantly reduced interference of biotic and abiotic factors [23,24].

Similarly to the results of Walters and Currey [25], Thai and Mexican basil did not differ significantly in total fresh weight, which was about twice that of Black and Mexican Purple, confirming the high variability in yield between these different varieties [3,11]. A similar trend was recorded for the total dry weight, with values ranging from 1.24 to 2.71 g pt^−1^ in the Mexican Purple and Thai, respectively (Table 1). Variations in yield may result from the interference of environmental factors, such as harvest time, cultural practices, and especially growing conditions that affect primary metabolism. In our study, basil grown in a floating raft system had a significantly higher average fresh yield than that obtained in the open field [9,26,27], confirming what Ciriello et al. [8,11] observed in three Genovese basil cultivars (Aroma 2, Eleonora, and Italiano Classico). The improved performance recorded in hydroponics could be attributable to unlimited availability of nutrients and water, low abiotic pressure, and high plant densities [11]. These results highlight the excellent adaptability of basil to hydroponic cultivation and the difficulty of comparing the production performance obtained with growth systems that differ in physical and technical characteristics, such as hydroponics and open field.

### 3.2. The Morphophysiological Traits That Make the Difference

Our results showed higher fresh yield of Thai compared to Genovese (Figure 1), which is the most cultivated basil variety worldwide. As shown in Table 1, this result is mainly attributable to the higher biomass allocation to the stem, with the highest values recorded for Thai and Mexican, followed by Genovese and Lemon, Black, and Mexican Purple. The higher stem biomass in Thai and Mexican resulted in the lowest leaf-to-stem ratio, in contrast to Mexican Purple, which recorded the highest value. These differences in the leaf-to-stem ratio between the above-mentioned basils are corroborated by the significant difference in plant height (Table 1), as reported by Walters and Currey [25] and Žlabur et al. [28] in similar works. Indeed, Thai was significantly taller than Genovese and Mexican Purple, a constitutive trait highlighting the strong influence of the genotype on this parameter. However, this morphological characteristic is detrimental for plant cultivation, since it could result in deleterious plant lodging, making it difficult to grow on floating panels [25]. Therefore, growers must select basil types with optimal leaf-to-stem ratio for the growing system, or new cultivation methods must be developed to limit this aspect. By this point of view, Mexican Purple was characterized by high leaf-to-stem ratio and lower height, resulting in higher yields per unit area (Table 1). These morphological traits make this variety very promising and possibly among the most suitable cultivars for high-density hydroponic cultivation.

Basil is grown primarily for its tender and fragrant leaves. Although Genovese differs significantly in leaf number but not leaf area from Thai, Mexican, and Lemon, it ensured higher fresh leaf production (Table 1). This result could be a consequence of the morphoanatomical differences between basil [17] and the different percentages of dry matter in the leaf (Table 1). The low dry matter of Genovese is a desired quality attribute for this type, which is intended for the industrial production of pesto sauce because a higher dry matter would prolong the processing time by promoting the establishment of oxidation that would lead to the subsequent blackening of the green sauce [8]. Except for Lemon, all basil had comparable total dry matter, and this difference could be the result of the phylogenetic distance between Lemon and the other basil varieties.

The evaluation of shelf life showed that after three days of storage, there were no differences between the varieties in terms of water loss, while at six days, Genovese showed significantly higher water loss than the average of the other varieties, suggesting the beginning of a more intense wilting phenomenon. More interestingly, the water loss in Thai and Mexican was significantly lower after nine days of storage than the average of the other varieties, which was correlated with a longer shelf life (Figure 2).

The results on the shelf life are consistent with previous reports, in which plants stored at 10 °C in the dark showed ~8 days of storability before showing symptoms of leaf deterioration [29]. It is worth noting that the lower water loss of Thai and Mexican varieties was correlated with higher levels of LMA of these varieties compared to the others. This indicates that a higher LMA could be a positive trait for fresh basil production, as it allows better control of water loss during storage. In addition, LMA is highly responsive to different environmental cues such as salinity, drought, and light intensity [30]. By this point of view, it could be interesting to evaluate whether it is possible to increase the shelf life of fresh basil by increasing the level of LMA induced by the application of controlled stresses and/or different light intensities/quality.

### 3.3. Mineral Profile, Pigments, and Secondary Metabolites Differ among Basil Varieties

According to Phippen and Simon [31], the lower production performance of Black and Mexican could be attributable to a high constitutive concentration of anthocyanins.

The accumulation of highly acylated anthocyanins would have a high metabolic cost, especially in the carbonic skeleton, drastically slowing the growth rate compared to green types [17]. In addition, anthocyanins are known to act as a screen for light, reducing incident light on the leaf [32]. Therefore, red leaf species generally show lower biomass accumulation due to lower CO_2_ assimilation rates and slower electron transport through the photosystems [33]. This aspect is confirmed by the gas exchange results, where Mexican Purple and Black, the two red-leaved varieties, showed the lowest photosynthetic activities compared to the other genotypes (Table 2), thus motivating their lower total dry weight compared to the other varieties. Significant differences between basil types were also observed for chlorophyll a, b, and total chlorophyll a concentration (Table 3). All basil types were characterized by a lower concentration of chlorophyll b than chlorophyll a. Although chlorophyll plays a crucial role in photosynthesis, the differences found in the six basil types did not significantly affect either production or physiology. Consistent with several authors [34,35], these results would confirm the vital role of genetics, since the differences recorded for chlorophyll concentration could result from the anatomical, morphological, and biometric differences observed. The above could also explain the differences recorded for the carotenoids (Table 3) whose accumulation and bioavailability, as reported by Kopsell et al. [36], depend primarily on genetic, biochemical, and physiological characteristics and are marginally affected by biotic and abiotic conditions. In addition to their role as accessory photosynthetic pigments, carotenoids play a crucial and complex biochemical function in the human body with potential beneficial effects [37]. A similar trend has been observed for the concentration of ascorbic acid (Figure 3), a bioactive molecule essential for human health, as it is directly involved in antioxidant and immune activity [38]. Similar to the reports of Muráriková and Neugebauerová [39] on seven basil cultivars, our results show that the ascorbic acid concentration ranged from 162.76 to 909.41 mg 100 g^−1^ fw. Although several studies have reported significantly higher ascorbic acid values in red cultivars [28,40], our results do not seem to confirm this trend. Although Mexican Purple reported the highest concentration (about six times that of Genovese), it differs significantly from Black.

Consistent with the results reported by Muráriková and Neugebauerová [39] and Ciriello et al. [8], the different varieties showed significant differences in leaf nitrate concentration (Figure 4). The values of this antinutrient typical of leafy vegetables were within the ranges reported in similar work by Muráriková and Neugebauerová [39], where the highest values were found in red cultivars. In contrast, our results partially disagreed with those of the previous authors. Although Black had the highest values, Mexican Purple was characterized by significantly lower nitrate concentrations than all basil types, but without differences from Lemon (Figure 4). As shown in Table 4, the complete mineral profile was significantly influenced by genetic material. For all types of basil, the most abundant mineral was K, while the least abundant mineral was Na. From this point of view, the mineral profile of basil was well balanced for these elements, considering that excessive Na intake has always been reported to predispose to hypertension, in contrast to K, which provides a positive reduction in blood pressure [37]. A specific condition was observed for the other mineral elements reported in Table 4. Specifically, for Thai and Mexican, the second mineral element was Ca, whose nutritional interest is primarily attributable to its role in promoting bone health (reducing the incidence of osteoporosis) [41]. The mineral profile of Lemon and Genovese, although characterized by a calcium concentration of 5.70 and 7.09 g kg^−1^ dw, the second mineral element of both was P (averaging 6.79 g kg^−1^ dw), another essential mineral for human skeletal health [42] and a key component of the photosynthetic process [9]. Ciriello et al. [43] and Carillo et al. [44] confirmed that in red basil and lettuce cultivars, chlorine was the second most abundant element in Black and Mexican Purple, compared to other green basil. Due to the known detrimental effects of Cl on plant primary metabolism [45,46,47], this result could partially explain the lower yields obtained by red basil.

## 4. Materials and Methods

### 4.1. Growth System, Plant Material, and Experimental Design

**Figure 5:**
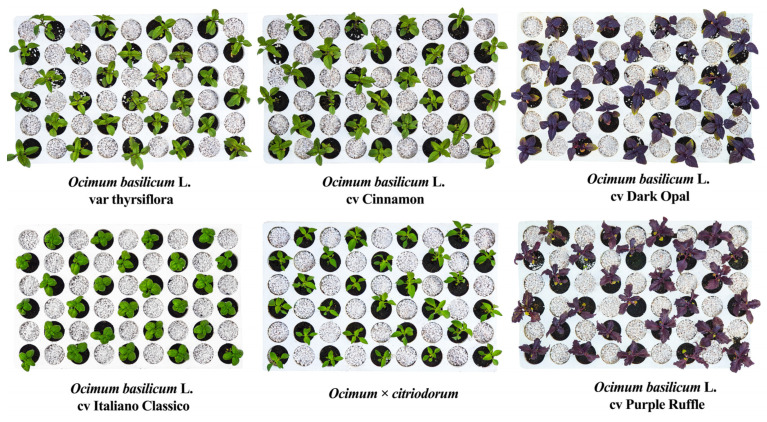
Illustrative picture of basil genotypes used in the experiment.

The present research aimed to compare the production and physiological performance of six different types of basil (Table 5 and Figure 5) grown in a floating raft hydroponic system (FRS). The experiment was carried out in a passively ventilated greenhouse at the University of Naples ‘Federico II’, Department of Agriculture (DIA) located in Portici (Naples, Italy; 40°48′ N, 14°20′ E, 29 m.a.s.l.). On 26 May 2021, Thai (*Ocimum basilicum* L. var thyrsiflora; Blumen, Milan, Italy), Mexican (*Ocimum basilicum* L. cv Cinnamon; Blumen, Milan, Italy), Black (*Ocimum basilicum* L. cv Dark Opal; Blumen, Milan, Italy), Genovese (*Ocimum basilicum* L. cv Italiano Classico; La Semiorto, Sarno, Italy), Lemon (*Ocimum* × citriodorum; Pagano Domenico & Figli Sementi, Scafati, Italy), and Mexican Purple (*Ocimum basilicum* L. cv Purple Ruffle; Pagano Domenico & Figli Sementi, Scafati, Italy) were transplanted in the phenological stage of 2–3 true leaves in polystyrene trays (52 × 33 cm) at a density of 158 plants m^−2^. For each basil type, the experimental unit consisted of one polystyrene tray containing 27 plants floating in a plastic thank filled with 35 L of nutrient solution (NS). Hoagland’s NS had the following macro and micronutrient composition: 14 mM N-NO_3_^−^, 1.75 mM S, 1.5 mM P, 3.0 mM K, 4.5 mM Ca, 1.5 mM Mg, 1.0 mM NH_4_^+^, 15 μM Fe, 9 μM Mn, 0.3 μM Cu, 1.6 μM Zn, 20 μM B, and 0.3 μM Mo. NS electroconductivity was 2.0 dS m^−1^, while pH was monitored and maintained at optimal values of 5.8. The oxygenation of the tanks was ensured with an immersion pump (Aquaball 60, Eheim, Stuttgart, Germany). The experimental design included four replications for each type of basil (24 experimental unit).

### 4.2. Collection, Processing, and Storage of Plant Samples

On 18 June (23 days after transplantation, DAT), the epigeal and hypogean parts of basil plants were sampled to determine biometric indices. Specifically, 15 plants per experimental unit were separated into leaves, stems, and roots to determine leaf number, fresh weight of leaves and stems, and root length. The leaf-to-stem ratio was then calculated. The leaf area was determined by digital image analysis with open-source ImageJ 1.52 h software (National Institutes of Health, MD, USA). A single, fully expanded leaf per each plant was sampled and scanned. The leaf area was measured with the same software used for total leaf area. Then, the scanned leaf was dried in a ventilated oven at 70 °C until constant weight. Leaf mass per area (LMA) was calculated as the ratio between leaf dry weight (g) and single leaf area (m^2^). All plant material collected (leaves, stems, and roots) was placed in a ventilated oven at 70 °C until a constant weight was reached to determine dry weights. The dry matter percentage was then determined. The dried plant material was finely ground with an MF10.1 Wiley laboratory mill and stored in sterile centrifuge tubes. Part of the aerial portion of the plants was immediately frozen at −80 ° C in liquid nitrogen for future qualitative analysis.

### 4.3. Shelf Life Evaluation

At harvest, 15 plants per variety have been weighted and sealed in plastic bags, selecting among the individuals showing no signs of damage. The plastic bags were stored at 10 °C in the darkness for nine days. After three (D3), six (D6), and nine (D9) days after the harvest, five plants per time point were weighted to evaluate the water loss percentage.

### 4.4. Determination of Leaf Gas Exchange and SPAD Index

The day before harvest (22 DAT), between 11:00 am and 2:00 pm, leaf gas exchange measurements were made on nine plants per experimental unit. The net rate of carbon dioxide assimilation (A_CO2_), stomatal resistance (gs), and transpiration rate (E) were determined using a portable gas exchange analyzer (Li-6400; LI-COR Biosciences, Lincoln, NE, USA) equipped with a 6.25 cm^2^ chamber. The airflow rate was established at 400 mL s^−1^ while environmental parameters such as carbon dioxide concentration, relative humidity, and photosynthetic photon flux density were established according to environmental values (365 ± 5 ppm, 700 ± 50 μmol m^−2^ s^−1^, 55 ± 5%, respectively). Instantaneous water use efficiency (WUEi) was calculated as A_CO2_/E.

On the same date as the leaf gas exchanges, the measurements of the SPAD index (chlorophyll meter SPAD-502 (Minolta Corp. Ltd., Osaka, Japan) were made on 20 young, fully expanded, healthy leaves of ten plants per experimental unit.

### 4.5. Determination of Mineral Concentration

The determination of the mineral profile (nitrate, potassium, phosphorus, calcium, magnesium, sodium, and chlorine) of dried and ground plant tissues was carried out by ion chromatography (ICS 3000, Thermo Fisher Scientific^TM^ Dionex^TM^, Sunnyvale, CA, USA) according to the method detailed by Formisano et al. [48]. Briefly, 0.25 g of finely ground and sieved dry sample was extracted in ultrapure water (Arium^®^ Advance EDI (Sartorius, Goettingen, Germany) by shaking the water bath (80 °C for 10 min; Julabo, Seelbach, Baden-Württemberg, Germany), centrifuged and injected into the ion chromatograph coupled with an electrical conductivity detector. Columns, precolumns, and self-healing suppressors were purchased from Thermo Scientific^TM^ Dionex^TM^ (Sunnyvale, CA, USA). Each treatment was analyzed in quadruplicate and the results were expressed as g kg^−1^ of dry weight (dw), except for nitrate, which was expressed as mg kg^−1^ fresh weight (fw).

### 4.6. Determination of the Concentration of Pigments and Ascorbic Acid

The determination of pigments (chlorophyll a, b, and carotenoids) and ascorbic acid was performed by spectrophotometry according to the methodology described by Lichtenthaler and Wellburn [49] and Kampfenkel et al. [50], respectively. Briefly, for pigment determination, an aliquot (0.5 g) of frozen plant sample was extracted in the dark in ammonia acetone for 15 min and then centrifuged for 5 min. From the extract obtained, the absorbance was read (Hach DR 4000; Hach Co, Loveland, CO, USA) at 647, 664, 470 nm to determine the chlorophyll a, b, and carotenoid concentrations, respectively. The results were expressed as mg g^−1^ fw. The total chlorophyll values were calculated as the sum of the relative chlorophylls (a and b).

To determine the total concentration of ascorbic acid, 0.4 g of fresh frozen plant tissues were ground with 0.8 mL of 6% trichloroacetic acid (Sigma Aldrich Srl, Milan, Italy) and incubated for 15 min at −20 °C. Then, an additional 1.2 mL of TCA was added to the obtained extract and centrifuged. The absorbance of the extract was read at a wavelength of 525 nm. The results were expressed as mg 100 g^−1^ fw.

All analyses were conducted in quadruplicate.

### 4.7. Statistics

All data are reported as mean ± standard error, *n* = 4 and were analyzed using IBM SPSS Statistics 26.0 software (SPSS Inc., Chicago, IL, USA). Mean effects were subjected to a one-way analysis of variance (ANOVA), and significance was determined using Tukey’s HSD test (*p* = 0.05). For the data regarding shelf life, the results of the ANOVA and the post-hoc test are reported within each time point (D3, D6, and D9 after harvest).

## 5. Conclusions

The solid genetic variability typical of the *Ocimum* genus could be an essential resource for hydroponic basil growers. A literature review revealed a lack of articles evaluating the production behavior of non-Genovese basil. The results of our experiment confirm the excellent adaptability of basil to hydroponic growing systems, showing significant differences in yield and morphophysiological traits. Genovese, Thai, and Mexican basil provided excellent yields, with the latter two types showing the best shelf life after nine days of cold storage attributable to a higher leaf mass area constituent. Lemon had the lowest nitrate value and the highest dry matter. On the other hand, despite being less productive, the red cultivars had high levels of levels of ascorbic acid and low levels of nitrate, especially in the Mexican purple. However, the lower production of red cultivars could easily be overcome by adopting ad hoc planting densities to fill the production gap with other cultivars. In light of the interesting and promising results, the comparison proposed in our work provides a solid basis for evaluating the strong genetic variability of basil in even greater detail. Understanding these aspects and insights into the nutritional quality of these basil types would confer important information to production sectors, such as breeding, processing industry, and pharma cosmetics.

## Figures and Tables

**Table 1:** Comparison of Thai, Mexican, Black, Genovese, Lemon, and Mexican Purple basil on biometric parameters.

Variables	Thai	Mexican	Black	Genovese	Lemon	Mexican Purple	Significance
	Mean ± Standard Error						
Leaf fw (g pt^−1^)	14.36 ± 0.31 b	14.02 ± 0.26 b	10.42 ± 0.37 c	16.52 ± 0.53 a	11.08 ± 0.70 c	9.60 ± 0.36 c	***
Stem fw (g pt^−1^)	16.59 ± 0.81 a	14.74 ± 0.66 a	6.14 ± 0.06 c	9.40 ± 0.38 b	9.48 ± 1.06 b	4.21 ± 0.18 c	***
Leaf-to-stem ratio fw	0.87 ± 0.03 d	0.96 ± 0.05 cd	1.70 ± 0.05 b	1.76 ± 0.03 b	1.20 ± 0.10 c	2.29 ± 0.06 a	***
Leaf dw (g pt^−1^)	1.61 ± 0.03 a	1.52 ± 0.08 ab	1.12 ± 0.04 cd	1.70 ± 0.08 a	1.32 ± 0.08 bc	1.00 ± 0.04 d	***
Stem dw (g pt^−1^)	1.10 ± 0.03 a	0.96 ± 0.07 ab	0.39 ± 0.01 de	0.57 ± 0.03 cd	0.77 ± 0.07 bc	0.24 ± 0.01 e	***
Root dw (g pt^−1^)	0.19 ± 0.01 ab	0.23 ± 0.02 ab	0.04 ± 0.01 c	0.27 ± 0.04 a	0.14 ± 0.03 b	0.03 ± 0.00 c	***
Total dw (g pt^−1^)	2.71 ± 0.06 a	2.48 ± 0.14 ab	1.52 ± 0.05 c	2.27 ± 0.11 ab	2.09 ± 0.15 b	1.24 ± 0.04 c	***
Leaf dm (%)	11.64 ± 0.27 ab	11.11 ± 0.18 bcd	11.37 ± 0.30 abc	10.24 ± 0.22 d	12.32 ± 0.30 a	10.44 ± 0.11 cd	***
Stem dm (%)	6.67 ± 0.14 b	6.51 ± 0.20 b	6.61 ± 0.19 b	6.04 ± 0.15 bc	8.15 ± 0.13 a	5.47 ± 0.13 c	***
Total dm (%)	8.77 ± 0.15 b	8.62 ± 0.40 b	9.16 ± 0.09 b	8.73 ± 0.19 b	10.18 ± 0.19 a	8.97 ± 0.07 b	***
LMA (g m^−2^)	33.31 ± 0.95 a	34.44 ± 1.72 a	25.60 ± 0.56 b	28.72 ± 0.98 b	26.60 ± 0.81 b	27.97 ± 0.68 b	***
Leaf area (cm^2^)	394.43 ± 10.01 ab	444.46 ± 24.32 a	402.42 ± 11.66 ab	418.00 ± 23.23 a	369.08 ± 41.25 ab	299.61 ± 16.57 b	**
Leaf number	51.50 ± 0.63 a	52.09 ± 1.48 a	33.25 ± 1.06 c	30.06 ± 0.76 c	46.63 ± 1.26 b	22.53 ± 0.91 d	***
Height (cm)	37.42 ± 0.68 a	33.61 ± 1.14 b	24.36 ± 0.33 d	27.09 ± 0.98 cd	28.25 ± 0.45 c	19.22 ± 0.29 e	***
Root length (cm)	49.75 ± 5.70	44.63 ± 2.68	42.81 ± 9.12	50.69 ± 3.25	41.88 ± 2.36	37.46 ± 2.83	n.s.

Data are mean values ± standard error, *n* = 4. Mean comparisons were performed by Tukey HSD test. Different letters indicate significant mean differences. n.s., **, and *** denote non-significant or significant effects at *p* ≤ 0.01 and 0.001, respectively. fw: fresh weight; dw: dry weight; dm: dry matter; LMA: leaf mass area; pt: plant.

**Table 2:** Comparison of Thai, Mexican, Black, Genovese, Lemon, and Mexican Purple basil on transpiration (E), stomatal conductance (gs), net CO_2_ assimilation rate (A_CO2_), and instantaneous water use efficiency (WUEi).

Treatment	E	gs	A_CO2_	WUEi
	mol H_2_O m^−2^ s^−1^	mol H_2_O m^−2^ s^−1^	μmol CO_2_ m^−2^ s^−1^	μmol CO_2_ mol^−1^ H_2_O
Thai	5.48 ± 0.23 ab	0.25 ± 0.01	22.71 ± 0.65 ab	4.16 ± 0.19 a
Mexican	5.70 ± 0.19 a	0.26 ± 0.01	24.40 ± 0.54 a	4.30 ± 0.23 a
Black	5.12 ± 0.07 ab	0.25 ± 0.01	10.83 ± 0.25 c	2.12 ± 0.05 b
Genovese	5.04 ± 0.08 b	0.23 ± 0.01	20.52 ± 0.64 b	4.08 ± 0.14 a
Lemon	5.26 ± 0.12 ab	0.25 ± 0.00	22.52 ± 0.54 ab	4.29 ± 0.13 a
Mexican Purple	5.42 ± 0.08 ab	0.26 ± 0.01	6.62 ± 0.27 d	1.22 ± 0.05 c
Significance	*	n.s.	***	***

Data are mean values ± standard error, *n* = 4. Mean comparisons were performed by Tukey HSD test. Different letters indicate significant mean differences. n.s., *, and *** denote nonsignificant or significant effects at *p* ≤ 0.05 and 0.001, respectively.

**Table 3:** Comparison of Thai, Mexican, Black, Genovese, Lemon, and Mexican Purple basil on leaf pigments.

Treatment	Chlorophyll a	Chlorophyll b	Total Chlorophyll	Carotenoids
	mg g^−1^ fw			
Thai	1.09 ± 0.03 ab	0.62 ± 0.04 ab	1.7 ± 0.07 ab	0.34 ± 0.02 a
Mexican	1.12 ± 0.03 ab	0.68 ± 0.04 ab	1.79 ± 0.07 ab	0.32 ± 0.02 ab
Black	1.02 ± 0.03 b	0.61 ± 0.04 b	1.62 ± 0.07 b	0.34 ± 0.01 a
Genovese	1.02 ± 0.00 b	0.57 ± 0.01 b	1.59 ± 0.01 b	0.34 ± 0.00 a
Lemon	1.18 ± 0.02 a	0.80 ± 0.04 a	1.97 ± 0.05 a	0.27 ± 0.01 b
Mexican Purple	1.09 ± 0.02 ab	0.75 ± 0.07 ab	1.83 ± 0.09 ab	0.30 ± 0.03 ab
Significance	**	**	**	*

Data are mean values ± standard error, *n* = 4. Mean comparisons were performed by Tukey HSD test. Different letters indicate significant mean differences. * and ** denote significant effects at *p* ≤ 0.05 and 0.01, respectively. fw: fresh weight.

**Table 4:** Comparison of Thai, Mexican, Black, Genovese, Lemon, and Mexican Purple basil mineral concentration.

Treatment	P	K	Ca	Mg	Na	Cl
	g kg^−1^ dw					
Thai	6.66 ± 0.29 ab	42.25 ± 1.46 c	8.58 ± 0.50 a	2.46 ± 0.13 ab	0.20 ± 0.03 d	2.86 ± 0.28 bc
Mexican	7.17 ± 0.22 ab	51.53 ± 0.72 a	8.23 ± 0.36 ab	2.82 ± 0.13 a	0.24 ± 0.05 cd	4.37 ± 0.46 b
Black	6.96 ± 0.09 ab	50.10 ± 0.23 ab	6.69 ± 0.27 c	2.26 ± 0.03 bc	0.31 ± 0.01 bcd	7.07 ± 0.32 a
Genovese	7.40 ± 0.21 a	52.17 ± 0.45 a	7.09 ± 0.16 bc	1.90 ± 0.08 cd	0.53 ± 0.01 a	2.49 ± 0.29 c
Lemon	6.18 ± 0.40 b	35.50 ± 2.66 d	5.70 ± 0.20 cd	1.67 ± 0.20 d	0.42 ± 0.04 ab	3.24 ± 0.28 bc
Mexican Purple	4.92 ± 0.23 c	43.45 ± 1.79 bc	4.71 ± 0.29 d	1.64 ± 0.08 d	0.36 ± 0.03 bc	7.21 ± 0.49 a
Significance	***	***	***	***	***	***

Data are mean values ± standard error, *n* = 4. Mean comparisons were performed by Tukey HSD test. Different letters indicate significant mean differences. *** denote significant effects at *p* ≤ 0.001. dw: dry weight.

**Table 5:** Comparison of morphological characteristics of Thai, Mexican, Black, Genovese, Lemon, and Mexican Purple basil.

Common Name	Scientific Name	Family	Leaf Characteristic	Plant Habitus
Thai basil	*Ocimum basilicum* L. var thyrsiflora	Lamiaceae	Small, pointed leaves with a very strong and intense aroma, slightly spicy between mint, clove, anise, and licorice.	Erect
Mexican basil	*Ocimum basilicum* L. cv Cinnamon	Lamiaceae	Oval, slightly serrated, and pointed leaves with a cinnamon aroma	Erect
Black basil	*Ocimum basilicum* L. cv Dark Opal	Lamiaceae	Oval, slightly serrated, and pointed leaves with a	Erect
Genovese basil	*Ocimum basilicum* L. cv Italiano Classico	Lamiaceae	Light green, slightly blistered, intensely fragrant leaves with no mint smell.	Erect
Lemon basil	*Ocimum × citriodorum*	Lamiaceae	Purple-colored, slightly boiling, intensely fragrant leaves without mint smell.	Erect
Mexican purple basil	*Ocimum basilicum* L. cv Purple Ruffle	Lamiaceae	Dark purple leaves curved along the midrib, serrated margin	Erect

## Data Availability

The data are contained within the article.
